# Evaluation of Sulfadiazine Degradation in Three Newly Isolated Pure Bacterial Cultures

**DOI:** 10.1371/journal.pone.0165013

**Published:** 2016-10-18

**Authors:** Sikandar I. Mulla, Qian Sun, Anyi Hu, Yuwen Wang, Muhammad Ashfaq, Syed Ali Musstjab Akber Shah Eqani, Chang-Ping Yu

**Affiliations:** 1 CAS Key Laboratory of Urban Pollutant Conversion, Institute of Urban Environment, Chinese Academy of Sciences, Xiamen, 361021, China; 2 Key Laboratory of Urban Environment and Health, Institute of Urban Environment, Chinese Academy of Sciences, Xiamen, 361021, China; 3 Graduate Institute of Environmental Engineering, National Taiwan University, Taipei 106, Taiwan; Purdue University, UNITED STATES

## Abstract

This study is aimed to assess the biodegradation of sulfadiazine (SDZ) and characterization of heavy metal resistance in three pure bacterial cultures and also their chemotactic response towards 2-aminopyrimidine. The bacterial cultures were isolated from pig manure, activated sludge and sediment samples, by enrichment technique on SDZ (6 mg L^-1^). Based on the 16S rRNA gene sequence analysis, the microorganisms were identified within the genera of *Paracoccus*, *Methylobacterium* and *Kribbella*, which were further designated as SDZ-PM2-BSH30, SDZ-W2-SJ40 and SDZ-3S-SCL47. The three identified pure bacterial strains degraded up to 50.0, 55.2 and 60.0% of SDZ (5 mg L^-1^), respectively within 290 h. On the basis of quadrupole time-of-flight mass spectrometry and high performance liquid chromatography, 2-aminopyrimidine and 4-hydroxy-2-aminopyrimidine were identified as the main intermediates of SDZ biodegradation. These bacteria were also able to degrade the metabolite, 2-aminopyrimidine, of the SDZ. Furthermore, SDZ-PM2-BSH30, SDZ-W2-SJ40 and SDZ-3S-SCL47 also showed resistance to various heavy metals like copper, cadmium, chromium, cobalt, lead, nickel and zinc. Additionally, all three bacteria exhibited positive chemotaxis towards 2-aminopyrimidine based on the drop plate method and capillary assay. The results of this study advanced our understanding about the microbial degradation of SDZ, which would be useful towards the future SDZ removal in the environment.

## Introduction

Sulfadiazine [4-amino-N-(2-pyrimidinyl)benzene sulfonamide, SDZ] is one of the most common sulfonamide antibiotics, utilized widely in animal husbandry to treat and inhibit bacterial diseases [1]. Conversely, treated animals have been reported to excrete unbroken SDZ with its metabolite(s) into the ecosystem through their excretory system [2]. For example, SDZ has been detected in the range of 0.3–198 mg kg^-1^ (dry matter of pig manure), depending on medication, dilution and age of the manure [3, 4] and also in slurry at high concentrations, almost 500 mg kg^-1^ [5]. Studies revealed that SDZ is mainly released into soil either by living systems and/or by application of contaminated manure from treated livestock as fertilizer on farming land [6]. Moreover, SDZ was also detected in Jiulong river and its estuary in south-east region of China [7]. The fate and effects of SDZ in manure during storage and into manure-applied soils have also been reported [8–10]. Hence, the persistence of SDZ in the environment is of great concern, because it might lead to severe damages to non-target organisms and thereby favors the increase of resistant bacteria and pose adverse health effects to human beings [11]. It is therefore necessary to study the metabolic fate of SDZ in the environment and its environmental degradation.

There are reports on sulfonamide antibiotics sorption activity, mobility, transformation as well as mineralization and the spread of antibiotic genes [12–17]. SDZ undergoes numerous transformation pathways and during the process can be inactivated (acetylation), altered into a less toxic molecule (hydroxylation), and/or to a highly hydrophilic metabolite with a lower molecular mass [18]. However, there are only limited reports on SDZ-degrading strains. To date, almost all independently isolated SDZ-degrading bacteria belonged to *Microbacterium* genus [19, 20]. On the other hand, 2-aminopyrimidine (major intermediate of SDZ) was not completely mineralized by strain SDZm4 [20]. Recently, degradation of 2-aminopyrimidine by *Terrabacter*-like bacterium has been studied and reported [21]. However, there is not much information available on both SDZ and 2-aminopyrimidine degradation by a single pure bacterium. Therefore, it is necessary to identify microorganisms with the ability to degrade SDZ and its major metabolite, 2-aminopyrimidine. In this study, we report the isolation and characterization of three different bacteria capable of degrading SDZ as well as 2-aminopyrimidine, and their resistance capabilities towards various heavy metals. In addition, we also report for the first time positive chemotactic response of *Paracoccus* sp. SDZ-PM2-BSH30, *Methylobacterium* sp. SDZ-W2-SJ40 and *Kribbella* sp. SDZ-3S-SCL47 towards 2-aminopyrimidine.

## Materials and Methods

### Chemicals and composition of the growth medium

Sulfadiazine was purchased from Sigma-Aldrich, Saint Louis, USA. Acetone, acetonitrile and methanol were purchased from Merck, Darmstadt, Germany. All other chemicals were of analytical grade or the highest grade available commercially. Stock solutions of SDZ, 2-aminopyrimidine and 2-amino-4-hydroxypyrimidine (1.00 g L^-1^) were prepared individually using methanol and stored in amber bottles at 4°C before use. The ammonium mineral salts (AMS) medium plus yeast extract (0.04%) supplemented with substrates like SDZ (5 mg L^-1^) and 2-aminopyrimidine (5 mg L^-1^) were prepared individually by the method described previously [22, 23]. The AMS medium was set to pH 7.00 (using 2 M NaOH or 2 M HCl), dispensed in 100 mL quantities into 250 mL Erlenmeyer flasks and finally sterilized by autoclaving for 20 min at 15 psi. Substrates (5 mg L^-1^) dissolved in methanol, were added to the autoclaved medium and after the evaporation of methanol, medium were inoculated.

### Isolation of microorganisms by enrichment culture technique

SDZ-degrading bacteria were isolated from pig manure (PM2), activated sludge (WWTPs, W2) and sediment (3S) samples, by enrichment on SDZ (6 mg L^-1^) as a sole source of carbon and energy. PM2 and 3S were collected from Maoming, China, whereas W2 was collected from Xiamen, China (approved by Xiamen Water Affairs Zhonghuan Sewage Treatment Co. Ltd.). Samples (5 g/ 5 mL) were suspended in 100 mL of sterile distilled water, mixed and filtered. The 5 mL of filtrates were added to 95 mL of sterile AMS medium (without yeast extract) in a 250 mL of Erlenmeyer flasks supplemented with SDZ (6 mg L^-1^) as a sole source of carbon and energy. The flasks were then incubated on a rotary shaker (150 rpm) at 30°C under dark condition for 30 days. After 30 days, 5 mL of the inoculums were transferred into fresh medium containing SDZ (6 mg L^-1^) and incubated for 30 days (until turbid) and the process was repeated four more times. The bacterial consortia were purified by serial dilution and pour plate method. AMS solid medium (without yeast extract) was prepared by adding 1.65% agar. A bacterial consortium was diluted serially and cultured on SDZ (6 mg L^-1^)-AMS agar plates. Different colonies obtained from bacterial consortium were further grown on AMS agar plates supplemented with SDZ (6 mg L^-1^). This process was performed several times to obtain pure bacterial cultures. The purified bacterial cultures designated as SDZ-PM2-BSH30 (PM2), SDZ-W2-SJ40 (W2) and SDZ-3S-SCL47 (3S) showed better growth on SDZ-AMS agar plates and therefore used in further studies.

### 16S rRNA gene sequence analysis for the identification of bacteria

For DNA, single colony of pure bacteria was transferred into 50 μL lysis buffer (Takara) in 1.5 mL tube, boiled at 80°C for 15 min and centrifuged (slowly). DNA sample (1 to 5 μL) was used for 16S rRNA gene amplification. The 16S rRNA gene was amplified by PCR using a pair of universal primers: 27F (5'-AGAGTTTGATCMTGGCTCAG-3') and 1492R (5'-GGTTACCTTTGTTACGACTT-3') [24]. PCR mixture contained 12.5 μL Premix Taq (Takara Biotechnology Co., Ltd, Dalian, China), 0.5 μM of each primer, approximately 1 μL of DNA template and sterile deionized water to make up the total reaction volume of 25 μL. PCR amplification was performed with a GenePro Thermal Cycler (Bioer, Hercules, China) and the amplification program consisted of: 1 cycle for 8 min at 94°C, followed by 37 cycles of 30 s at 94°C, 30 s at 55°C, 1.5 min at 72°C; and finally 1 cycle for 5 min at 72°C. The purified PCR products of 16S rRNA genes were sequenced and analyzed by comparing and aligning with relative gene sequences available in Ez-Taxon and GenBank data libraries using BLAST-n and CLUSTAL-W program. The phylogenetic trees were constructed on the basis of maximum-likelihood method using the Molecular Evolutionary Genetics Analysis software (MEGA6) [25].

### Growth and degradation of SDZ and 2-aminopyrimidine

To monitor the effects of initial concentrations of SDZ and 2-aminopyrimidine on the growth of *Paracoccus* sp. SDZ-PM2-BSH30, *Methylobacterium* sp. SDZ-W2-SJ40 as well as *Kribbella* sp. SDZ-3S-SCL47 and their degradation, the microorganisms were grown on 100 mL of AMS-yeast extract (0.04%) in 250 mL Erlenmeyer flasks supplemented with appropriate concentration of SDZ (2–8 mg L^-1^) as well as 2-aminopyrimidine (3–7 mg L^-1^). The bacterial growth was measured at 600 nm by UV-spectrophotometer (UV-5200 Spectrophotometer). For SDZ (5 mg L^-1^) and 2-aminopyrimidine (5 mg L^-1^) degradation studies, the samples collected at regular intervals were centrifuged (7000 × *g*) and then analyzed by high performance liquid chromatography (HPLC). Detailed description is provided in Supporting Information. Uninoculated culture flasks with the same amount of SDZ, 2-aminopyrimidine, SDZ plus sterilized individual bacterial cells and 2-aminopyrimidine plus sterilized individual bacterial cells were served as controls.

### Identification of metabolites

The metabolites of SDZ (5 mg L^-1^) in cell-free filtrates of *Paracoccus* sp. SDZ-PM2-BSH30, *Methylobacterium* sp. SDZ-W2-SJ40 and *Kribbella* sp. SDZ-3S-SCL47 were identified by quadrupole time-of-flight-mass spectrometry (MS) (Q-/TOF-MS, SI). The cell-free filtrates were collected at regular intervals. The same individual bacterial cultures supernatant without SDZ were used as negative controls, and uninoculated controls containing SDZ (5 mg L^-1^) were included as well. The collected samples were centrifuged (7000 × *g*) for 20 min, adjusted to pH 2.0 with 2 M HCl and then extracted with ethyl acetate. The extracted samples were reconstituted in methanol (500 μl), filtered and analyzed by Q/TOF-MS.

### SDZ-degrading bacteria’s resistance to heavy metals and their minimum inhibition concentration (MIC)

Stock solutions (1000 mg L^-1^) of the targeted heavy metals were prepared by dissolving an accurately weighted amount of their corresponding salts into Milli-Q water. Dissolved salts involved (CdCl_2_)_2_.5H_2_O, CoCl_2_.6H_2_O, CuCl_2_.2H_2_O, ZnCl_2_, NiCl_2_.6H_2_O, Pb(NO_3_)_2_ and K_2_Cr_2_O_7_. Stock solutions were stored at 4°C.

The SDZ-degrading bacterial cultures including *Paracoccus* sp. SDZ-PM2-BSH30, *Methylobacterium* sp. SDZ-W2-SJ40 and *Kribbella* sp. SDZ-3S-SCL47 were tested for the resistance to various heavy metals like copper (Cu), cadmium (Cd), chromium (Cr), cobalt (Co), lead (Pb), nickel (Ni) and zinc (Zn) using sucrose-minimal salts low-phosphate (SLP) medium whose composition is as follows; sucrose 0.9%, (NH_4_)_2_SO_4_, 0.1%, K_2_HPO_4_, 0.05%, MgSO_4_, 0.05%, NaCl, 0.01%, yeast extract, 0.04% and CaCO_3_, 0.05%. The pH of the medium was kept around 7.05–7.10 [26]. The bacterial cultures grown on SDZ were placed onto the agar plates of SLP supplemented with individual heavy metals. The cell concentration of SDZ-PM2-BSH30, SDZ-W2-SJ40 and SDZ-3S-SCL47 were 3.3×10^7^, 3.7×10^7^ and 3.4×10^7^ cfu/mL, respectively. Inoculated plates were incubated at 30°C for 72 h. SLP agar plate without heavy metal was used as a positive control. The lowest heavy metal concentration that prevented growth was recorded as the minimum inhibitory concentration (MIC).

### Chemotaxis towards 2-aminopyrimidine

The chemotactic response of *Paracoccus* sp. SDZ-PM2-BSH30, *Methylobacterium* sp. SDZ-W2-SJ40 and *Kribbella* sp. SDZ-3S-SCL47 towards 2-aminopyrimidine was examined qualitatively (drop plate assay) and quantitatively (capillary assay) by following procedures described earlier [27, 28]. For drop plate method, the bacterial cells of SDZ-PM2-BSH30, SDZ-W2-SJ40 and SDZ-3S-SCL47 were grown in LB supplemented with SDZ (5 mg L^-1^). The cultures were harvested at mid-log phase (O.D_600_ between 0.60–0.70) by centrifugation at 4000 × *g* for 10 min and pellets were washed twice with phosphate buffered saline (PBS), re-suspended in drop plate assay medium (AMS with 0.3% bacto agar) and transferred into petri-plates (96 mm). Pinch of 2-aminopyrimidine was placed in the middle of plates and then incubated at 25°C. The chemotactic response was observed after 5–8 h of incubation. For quantitative capillary method, the optimum concentration of 2-aminopyrimidine was determined by carrying out at various concentrations of 2-aminopyrimidine (2–8 mg L^-1^). Initially, 10 μL of glass capillaries were filled with the desired amount of 2-aminopyrimidine (in chemotaxis buffer) and the suction end was closed by sterile agarose gel. The control capillaries were without any chemotactic compound. Capillaries were individually inserted into micro centrifuge tubes (separately) and having a suspension (10^10^ to 10^11^ cells mL^-1^) of SDZ-PM2-BSH30, SDZ-W2-SJ40 as well as SDZ-3S-SCL47 and were incubated at 25°C for 40 min. The solutions of capillaries were then serially diluted and spread onto LB agar plate, followed by determination of CFUs count after 48 h incubation at 37°C. Chemotaxis index (ratio of the number of CFUs produced from capillary supplemented with test substrate to CFUs produced from a control capillary) was used to quantify the chemotactic response. Aspartate was used as the positive control.

## Results

### Isolation and characterization of microorganisms

**Fig 1:**
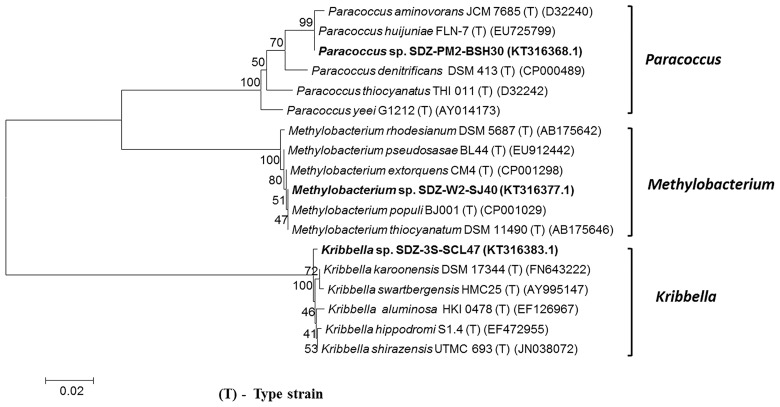
Phylogenetic relationships established by the maximum-likelihood method (using MEGA6 software) based on 16S rRNA gene sequences of isolated bacterial strains (SDZ-PM2-BSH30, SDZ-W2-SJ40, and SDZ-3S-SCL47). Scale bar, no. of nucleotide changes/sequence position. The number at nodes shows the bootstrap values obtained with 1,000 resampling analyses.

SDZ (6 mg L^-1^) degrading microorganisms were isolated from PM2, W2 and 3S samples by enrichment culture technique. On the basis of 16S rRNA gene sequence analysis, SDZ-PM2-BSH30, SDZ-W2-SJ40 and SDZ-3S-SCL47 were identified as the member of genera of *Paracoccus*, *Methylobacterium* and *Kribbella*, respectively. Phylogenetic tree was constructed by 16S rRNA gene sequences in comparison with other related bacteria and are shown in Fig 1. The bacteria, *Paracoccus* sp. SDZ-PM2-BSH30 and *Methylobacterium* sp. SDZ-W2-SJ40 belong to Gram-negative whereas *Kribbella* sp. SDZ-3S-SCL47 belongs to Gram-positive bacteria. The 16S rRNA gene sequence of SDZ-PM2-BSH30, SDZ-W2-SJ40 and SDZ-3S-SCL47 were deposited in NCBI under accession number KT316368, KT316377 and KT316383, respectively.

### Degradation of SDZ and 2-aminopyrimidine by three pure bacteria

**Fig 2:**
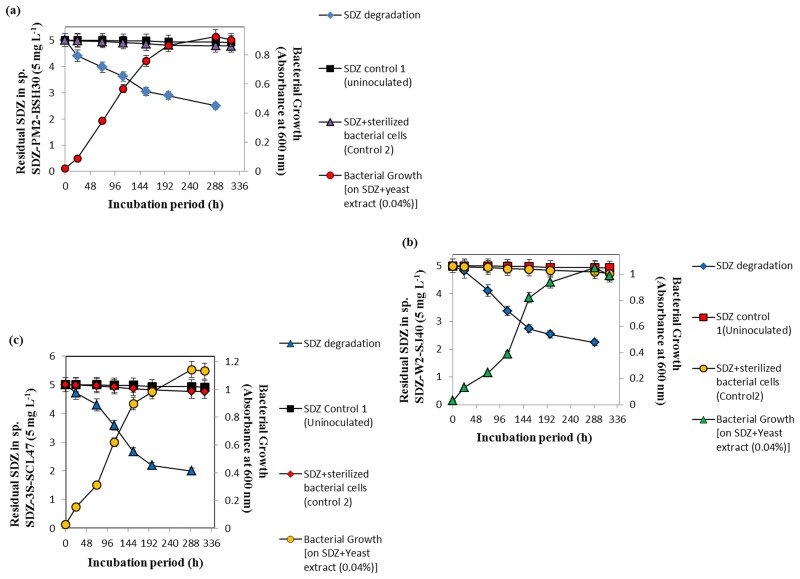
SDZ (5 mg L^-1^) degradation by SDZ-PM2-BSH30 (a), SDZ-W2-SJ40 (b) and SDZ-3S-SCL47 (c). Error bar represents the standard deviation of the triplicates.

**Fig 3:**
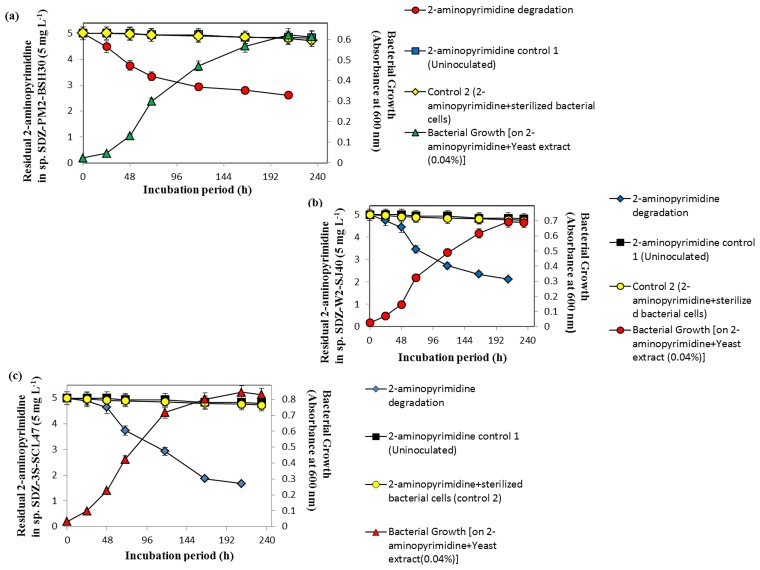
2-Aminopyrimidine (5 mg L^-1^) degradation by SDZ-PM2-BSH30 (a), SDZ-W2-SJ40 (b) and SDZ-3S-SCL47 (c). Error bar represents the standard deviation of the triplicates.

The individual bacterial cultures growth (*Paracoccus* sp. SDZ-PM2-BSH30, *Methylobacterium* sp. SDZ-W2-SJ40 and *Kribbella* sp. SDZ-3S-SCL47) in AMS medium plus 0.04% of yeast extract and SDZ (5 mg L^-1^) were monitored and are shown in the Fig 2. The strains SDZ-PM2-BSH30, SDZ-W2-SJ40 and SDZ-3S-SCL47 degraded SDZ (5 mg L^-1^) up to 50.0, 55.2 and 60.0%, respectively within 290 h. No further SDZ degradation was observed even after incubating for prolonged periods (>320 h). In addition, the bacteria were also able to grow on yeast extract (0.04%)-AMS medium in the presence of 2-aminopyrimidine and 2-amino-4-hydroxypyrimidine at an initial concentration of 5 mg L^-1^, respectively. Furthermore, 2-aminopyrimidine (5 mg L^-1^) was degraded up to 47.8, 57.6 and 66.4% by SDZ-PM2-BSH30, SDZ-W2-SJ40 and SDZ-3S-SCL47, respectively within 210 h (Fig 3). On the other hand, in control experiments, SDZ and 2-aminopyrimidine at 5 mg L^-1^ were adsorbed by killed cells at negligible amount (Figs 2 and 3). Hence, the results in this study clearly demonstrated the degradation of SDZ and 2-aminopyrimidine by *Paracoccus* sp. SDZ-PM2-BSH30, *Methylobacterium* sp. SDZ-W2-SJ40 and *Kribbella* sp. SDZ-3S-SCL47 (Figs 2 and 3).

### Identification of metabolites

The identification of SDZ metabolites was conducted by high resolution MS and HPLC. S1 Fig provides the mass spectrometry of the culture supernatant of SDZ-3S-SCL47. The signal of *m/z* at 96.056 was identified as [2-aminopyrimidine+H]^+^, which was in accordance to Tappe’s work [21]. The signal of *m/z* at 111.05 was identified as 2-amino-4-hydroxypyrimidine. 2-Amino-4-hydroxypyrimidine was confirmed by HPLC chromatogram (S2 Fig). The retention time of the 2-amino-4-hydroxypyrimidine standard solution was 2.29 min (S2b Fig), while in the culture supernatant of SDZ-3S-SCL47, a peak was observed at similar time, which suggested the presence of 2-amino-4-hydroxypyrimidine (S2a Fig). The two metabolites were also observed in the culture supernatants of SDZ-PM2-BSH30 and SDZ-W2-SJ40 (data not shown).

### Characterization of various heavy metal resistance in three pure bacteria

The SDZ-degrading bacteria including *Paracoccus* sp. SDZ-PM2-BSH30, *Methylobacterium* sp. SDZ-W2-SJ40 and *Kribbella* sp. SDZ-3S-SCL47 were challenged to heavy metals for the determination of the MIC values. All three newly isolated pure bacteria showed resistance to Cu, Cd, Cr, Co, Pb, Ni and Zn (Table 1). SDZ-PM2-BSH30 showed high MIC to Pb (214 mg L^-1^) whereas SDZ-W2-SJ40 showed high MIC to Pb (168 mg L^-1^), Co (204 mg L^-1^) as well as Zn (146 mg L^-1^) and SDZ-3S-SCL47 showed high MIC to Pb (220 mg L^-1^), Ni (169 mg L^-1^) and Zn (216 mg L^-1^) (Table 1). The bacteria SDZ-PM2-BSH30 showed least MIC to Cr (8 mg L^-1^), Cd (16 mg L^-1^) and Ni (14 mg L^-1^) whereas SDZ-W2-SJ40 showed least MIC to Cr (24 mg L^-1^) and SDZ-3S-SCL47 showed least MIC to Cd (9 mg L^-1^) (Table 1).

**Table 1:** Minimum inhibitory concentration of heavy metal for bacterial isolates.

Bacteria	Minimum inhibitory concentration of heavy metals (mg L^-1^)						
	Pb	Cu	Co	Cr	Cd	Ni	Zn
SDZ-PM2-BSH30	214	31	26	8	16	14	47
SDZ-W2-SJ40	168	47	204	24	122	108	146
SDZ-3S-SCL47	220	42	115	37	9	169	216

### Chemotaxis of SDZ-PM2-BSH30, SDZ-W2-SJ40 and SDZ-3S-SCL47 towards 2-aminopyrimidine

**Fig 4:**
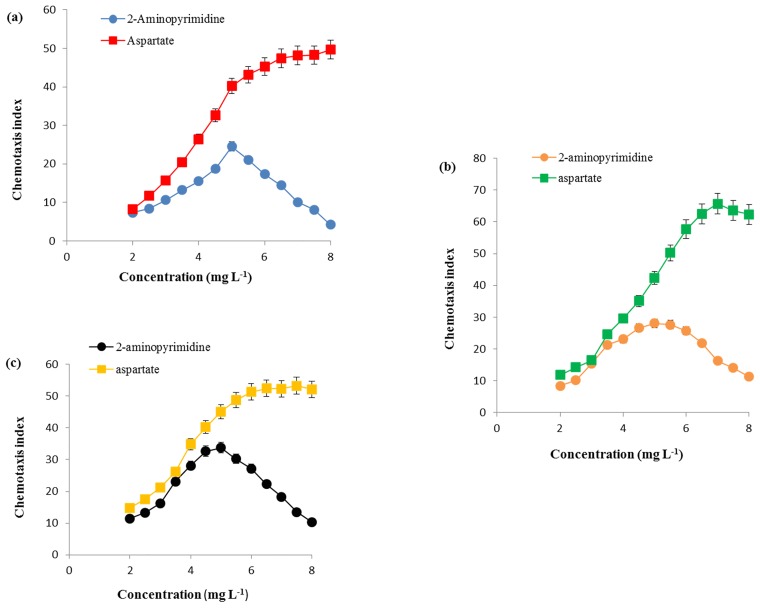
Quantitation of the chemotactic response and determination of optimal response concentration for *Paracoccus* sp. SDZ-PM2-BSH30 (a), *Methylobacterium* sp. SDZ-W2-SJ40 (b) and *Kribbella* sp. SDZ-3S-SCL47 (c) towards 2- aminopyrimidine using capillary assay. Error bar represents the standard deviation of the triplicates.

**Fig 5:**
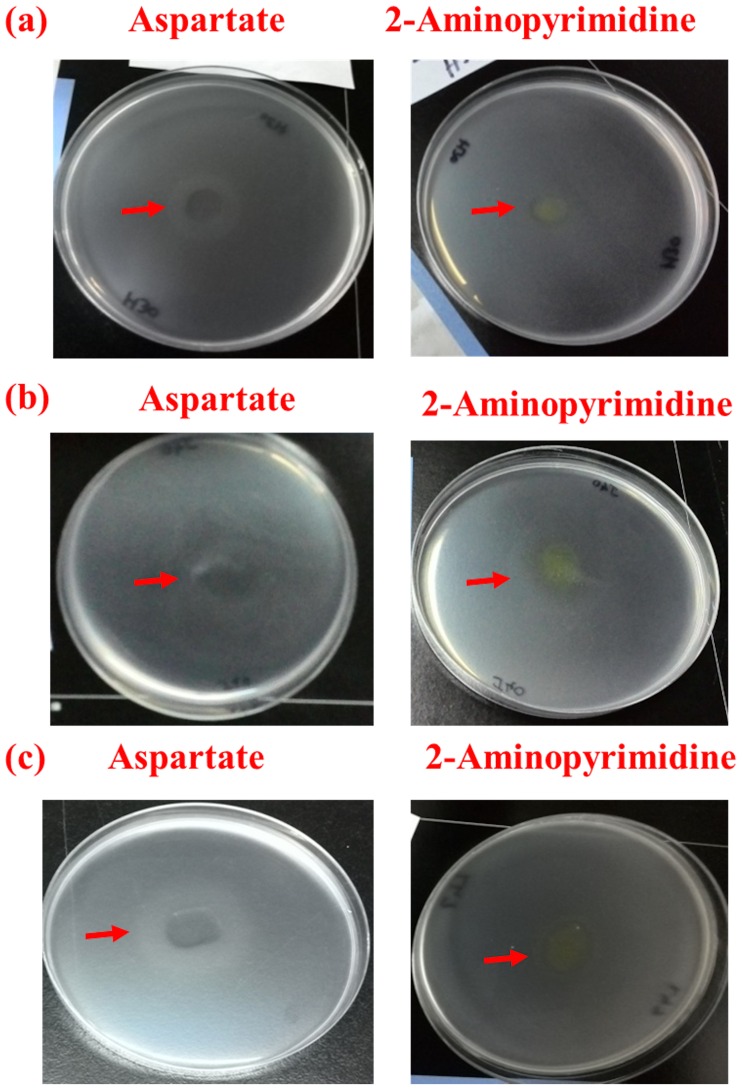
Chemotaxis of bacteria like SDZ-PM2-BSH30 (a), SDZ-W2-SJ40 (b), and SDZ-3S-SCL47 (c) toward 2-aminopyrimidine. The bacterial cells were grown on SDZ and tested on 2-aminopyrimidine. Results were obtained by drop plate assays. The assays were performed in triplicate and the representative plates are shown here. Aspartate was used as the positive control.

The chemotactic behavior of *Paracoccus* sp. SDZ-PM2-BSH30, *Methylobacterium* sp. SDZ-W2-SJ40 and *Kribbella* sp. SDZ-3S-SCL47 towards 2-aminopyrimidine was also studied by capillary assay and drop plate method. In capillary assay, it was observed that the cells of SDZ-PM2-BSH30, SDZ-W2-SJ40 and SDZ-3S-SCL47 were chemotactic towards 2-aminopyrimidine at an optimum concentration of 5 mg L^-1^ with chemotaxis index of 24.52, 28.10 and 33.76, respectively. As shown in the Fig 4, the chemotaxis index values for all three bacteria increased with increasing concentration until the optimal concentration. Further increase in concentration of 2-aminopyrimidine led to sharp decline of chemotaxis index values in SDZ-PM2-BSH30, SDZ-W2-SJ40 and SDZ-3S-SCL47. Aspartate was a positive control and there was not much decrease of chemotaxis index values in all three bacterial cultures (Fig 4). Drop plate assays showed the formation of bacterial ring around the crystals of 2-aminopyrimidine after incubation between 4–6 h (Fig 5). This is well supported to the data of capillary assay.

## Discussion

In this study, the screening of SDZ-degrading bacteria by enrichment method from various samples like PM2, W2 and 3S allowed to select the potential isolates with high survivability to various heavy metals and chemotactic towards 2-aminopyrimidine. Overall, three newly isolated pure bacteria were studied and compared for their degradation capability on both SDZ and 2-aminopyrimidine at 5 mg L^-1^. The bacterial clutters like SDZ-W2-SJ40 and SDZ-3S-SCL47 showed higher degradation rate of SDZ (5 mg L^-1^). None of these bacteria were previously reported for the degradation of both SDZ and 2-aminopyrimidine. In addition, SDZ-W2-SJ40 and SDZ-3S-SCL47 showed higher degradation of 2-aminopyrimidine than SDZ-PM2-BSH30. There are reports on addition of yeast extract during the degradation of toxic or inhibitory pollutant(s) by pure bacteria and/or bacterial consortia [22, 29]. Previous studies reported that the addition of yeast extract increases the growth of microorganisms thereby enhancing the toxic pollutant degradation [29, 30]. In the present study, addition of 0.04% of yeast extract helped to enhance the bacterial growth and achieve faster degradation of SDZ, but the three strains could also degrade SDZ without addition of 0.04% of yeast extract (data not shown).

**Fig 6:**
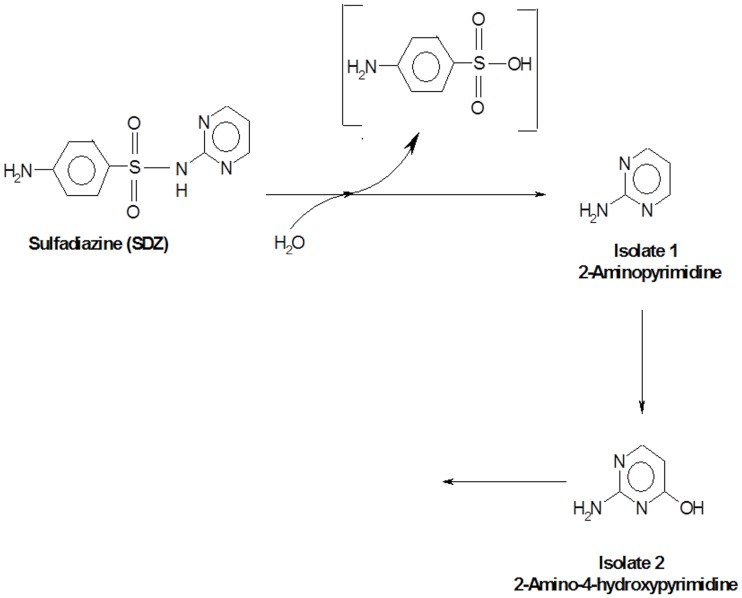
A proposed pathway of SDZ degradation by *Kribbella* sp. SDZ-3S-SCL47.

On the basis of identified metabolites and also degradation of 2-aminopyrimidine in SDZ-3S-SCL47, a pathway for the degradation of SDZ in *Kribbella* sp. SDZ-3S-SCL47 was proposed (Fig 6). Similarly, in other two bacteria like SDZ-PM2-BSH30 and SDZ-W2-SJ40, SDZ was also transformed into 2-aminopyrimidine which was further converted to 2-amino-4-hydroxypyrimidine (data not shown). No further metabolites were observed in all three pure bacterial cultures. Moreover, all three bacteria showed the ability to degrade 2-aminopyrimidine. The initial step of SDZ catabolism involved ipso-hydroxylation to form 2-aminopyridine whereas other by-products were not detected in this case. Similarly, in *Microbacterium lacus* strain SDZm4, SDZ was transformed into 2-aminopyrimidine [20]. The metabolite 2-aminopyridine was observed not only by microbial process, but also detected during photolysis, electrochemical oxidation and sorption experiments by soils [1, 18, 31, 32]. Furthermore, in strain 2APm3, 2-aminopyrimidine was metabolized into two by-products with one of that identified as 2-amino-4-hydroxypyrimidine [21]. Similarly, in this study, the bacteria degraded 2-aminophyrimidine into 2-amino-4-hydroxypyrimidine.

In addition, *Paracoccus* sp. SDZ-PM2-BSH30, *Methylobacterium* sp. SDZ-W2-SJ40 and *Kribbella* sp. SDZ-3S-SCL47 were resistant to various heavy metals. *Paracoccus* sp. SDZ-PM2-BSH30 showed high resistance to Pb, *Methylobacterium* sp. SDZ-W2-SJ40 showed high resistance to Pb, Co and Zn whereas *Kribbella* sp. SDZ-3S-SCL47 showed high resistance towards Pb, Ni and Zn. These high levels of heavy metal resistance could be beneficial for living and adjusting of these microbial strains in severe heavy metal polluted environment. There are reports on various organisms capable of resisting different heavy metals [26, 33–37]. Future study using molecular biology techniques, possibly will help to understand the mechanism of heavy metal resistance in SDZ-degrading pure bacteria.

Microbial chemotactic response was identified as an important phenomenon of bacteria to different toxic pollutants and thus, such bacteria were useful for the bioremediation of toxic pollutants in the environment [38]. Hence, it is suggested that chemotactic response can increase the biodegradation through improving the availability of substrate to support the development of mixed bacteria with the capabilities of biodegradation [27, 38, 39]. There are reports on various bacteria having the ability of chemotactic response towards various toxic pollutants such as nitroaromatics, organophosphate, chloroaromatics and aminobenzoates etc. [27, 40, 41]. In this study, SDZ-degrading bacteria such as *Paracoccus* sp. SDZ-PM2-BSH30, *Methylobacterium* sp. SDZ-W2-SJ40 and *Kribbella* sp. SDZ-3S-SCL47 showed chemotaxis towards 2-aminopyrimidine, which these bacteria can degrade metabolically or co-metabolically. The chemotactic response (capillary method) results in this study were similar to the results observed in *Pseudomonas* sp. strain BUR11 (chemotaxis towards organophosphate compound) [40]. However, it is necessary to investigate at genomic level for better understanding of this chemotactic activity in all of these three pure bacterial cultures, since this feature is a novel and characteristic to our SDZ-degrading bacteria. From previous reports, it has been observed that the bacterial chemotactic response towards various aromatic compounds proceeds via two different mechanisms (metabolism dependent and metabolism independent). There are reports on various microorganisms, which showed metabolism dependent chemotactic response to different compounds [27, 42, 43]. Similarly, metabolism independent chemotaxis has been also reported for different bacterial strains towards various toxic chemicals [41, 44, 45]. However, we are not sure whether this chemotaxis behavior is metabolically independent and/or dependent [40, 42], although results presented in this study demonstrated that all three bacteria had the ability to degrade both SDZ and 2-aminopyrimidine. More experiments related to chemotaxis behavior of these bacteria for those substrates which these strains were not able to metabolize and/or transform will be needed to clarify the chemotactic mechanism.

## Conclusions

In this study, we have isolated three different bacteria, including *Paracoccus* sp. SDZ-PM2-BSH30, *Methylobacterium* sp. SDZ-W2-SJ40 and *Kribbella* sp. SDZ-3S-SCL47 by enrichment on SDZ. In all three bacterial cultures, SDZ was initially transformed into 2-aminopyrimidine which was further converted to 2-amino-4-hydroxypyrimidine. Furthermore, these pure bacterial cultures were also capable of degrading 2-aminopyrimidine. The MIC results revealed that *Paracoccus* sp. SDZ-PM2-BSH30, *Methylobacterium* sp. SDZ-W2-SJ40 and *Kribbella* sp. SDZ-3S-SCL47 were able to resist various heavy metals. In addition, all three bacteria showed chemotaxis towards 2-aminopyrimidine. Hence, these microorganisms showed potential use in future bioremediation of SDZ in the environment.

## Supporting Information

S1 Fig Q-/TOF-MS spectrum of a culture supernatant of SDZ catabolism in *Kribbella* sp. SDZ-3S-SCL47. (TIF)

S2 Fig HPLC Chromatogram of 2-aminopyrimidine catabolism in *Kribbella* sp. SDZ-3S-SCL47 (a) with authentic 2-amino-4-hydroxypyrimidine (b). (TIF)

S1 Method Analytical Methods. (DOC)
