# Supplementary figures and images for: Evaluation of Sulfadiazine Degradation in Three Newly Isolated Pure Bacterial Cultures
Source: PLoS One. 2016 Oct 18;11(10):e0165013. doi: 10.1371/journal.pone.0165013 (PMC5068754; doi:10.1371/journal.pone.0165013)

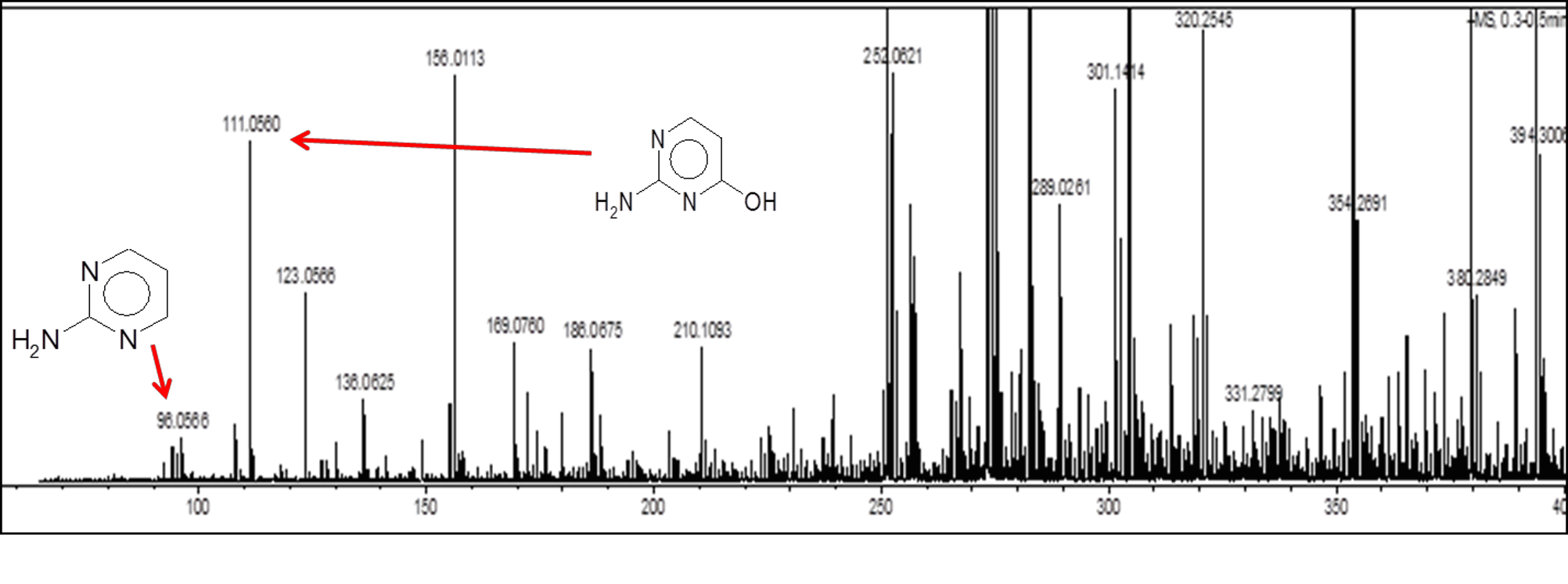

Supplement: S1 Fig — (TIF) [file pone.0165013.s001.tif]

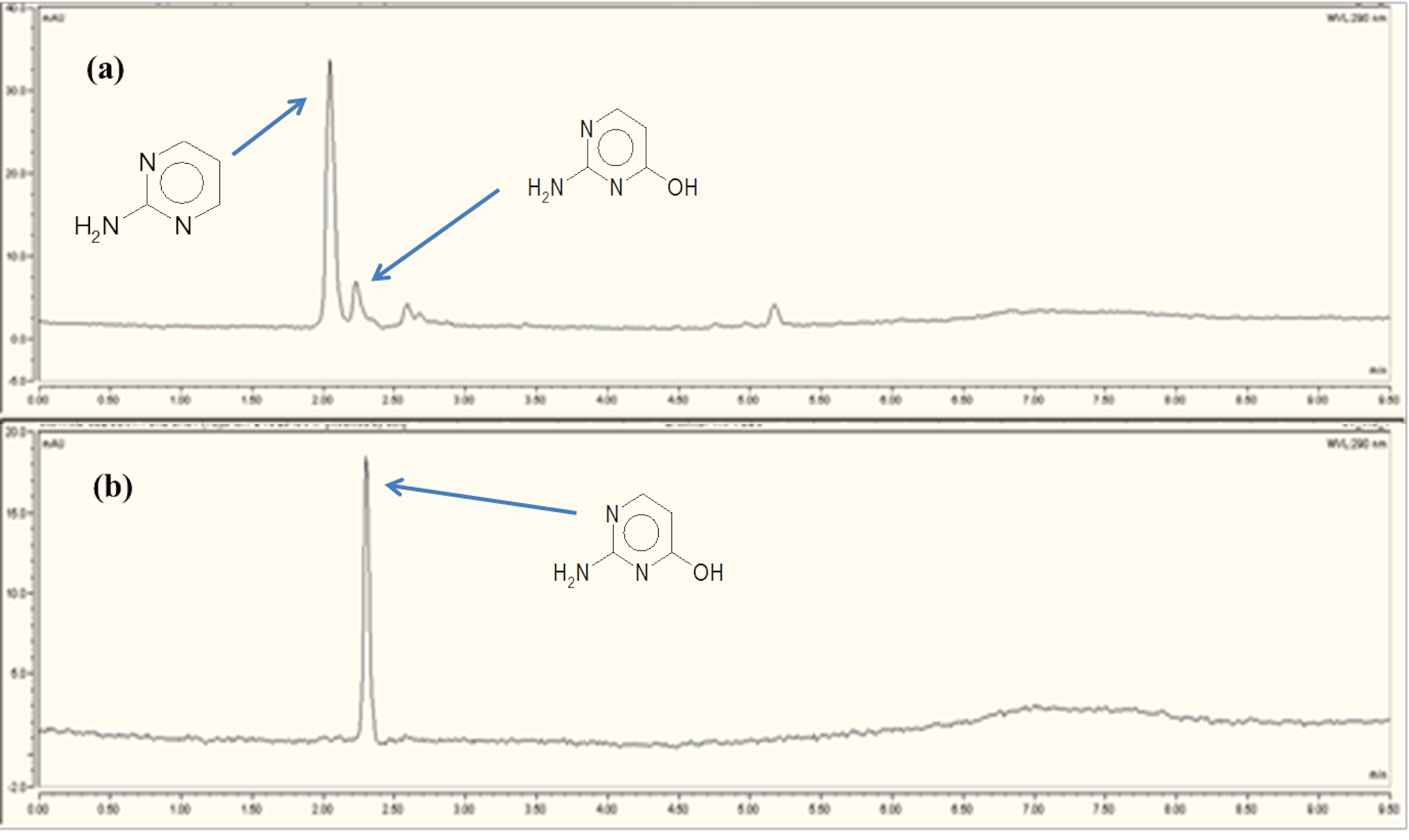

Supplement: S2 Fig — (TIF) [file pone.0165013.s002.tif]
