# Supplementary material for: Evaluation of Sulfadiazine Degradation in Three Newly Isolated Pure Bacterial Cultures
Source: PLoS One. 2016 Oct 18;11(10):e0165013. doi: 10.1371/journal.pone.0165013 (PMC5068754; doi:10.1371/journal.pone.0165013)
Supplement: S1 Method — (DOC) [file pone.0165013.s003.doc]

**Analytical Methods**

SDZ and 2-amynopyrimidine concentrations were determined by HPLC (Dionex Ultimate 3000, USA) equipped with a UV detector (254 nm and 290 nm, respectively). The separation was performed on LabChrom C_18_ (5 µm, 4.6 mm × 250 mm, Hitachi) using pure methanol (MeOH) and mixture of water-methanol (49:1) buffered with 1 mL L^-1^ phosphoric acid (25%), as the mobile phase at a flow rate of 1.2 mL min^-1^. The elution gradient was as follows: 20% MeOH for 1 min and increased to 80% MeOH up to 5 min, held at 80% MeOH for 2 min, decreased back to 20% MeOH over 0.2 min, and held at 20% MeOH for 2.3 min. The metabolites of SDZ (5 mg L^-1^) in cell-free ﬁltrates of organisms were identified by Q-TOF-MS (Bruker, Germany). MS was operated in electrospray ionization (ESI) positive mode, with a capillary voltage of 4.5 kV and collision energy of 8 eV.
